# 00004 Accuracy of the PREP2 algorithm for predicting Three Month Upper Limb Functional Capacity within a United States population of Persons with Stroke

**DOI:** 10.1017/cts.2021.689

**Published:** 2021-03-30

**Authors:** Jessica Barth, Kimberly Waddell, Marghuretta D. Bland, Catherine E. Lang

**Affiliations:** Washington University in St. Louis

## Abstract

ABSTRACT IMPACT: Evaluate the accuracy of applying a predictive algorithm using clinical measures only in persons with stroke in the US. OBJECTIVES/GOALS: PREP2 is an algorithm, that predicts UL functional capacity at 3 months post stroke from measures taken within the first week.(1, 2) Despite its accuracy and ease of use, challenges arise of applying PREP2 in the US. The objective of this study was to evaluate the accuracy of PREP2 using only clinical measures in persons with stroke in the US. METHODS/STUDY POPULATION: Individuals with first-ever stroke were recruited from a local hospital and followed longitudinally, as part of an ongoing observational cohort. Variables captured within two weeks of stroke and entered into the algorithm were: age, SAFE score(1-3) and NIH Stroke Scale(4) total score. The algorithm classifies individuals into one of four expected categories: excellent, good, limited, or poor. The dependent variable was the predicted category of UL functional capacity as defined by ranges of the 3-month Action Research Arm Test score.(5) Accuracy, specificity, sensitivity, positive predictive value (PPV), and negative predictive value (NPV) of the algorithm, were calculated using a 4x4 contingency table. Other statistics analyzed include demographic characteristics and a weighted kappa for the algorithm. RESULTS/ANTICIPATED RESULTS: Data from 49 individuals were analyzed (57% male, 88% ischemic stroke, age = 65±8.56 years). Expected categorization matched observed categorization in 29/49 subjects, with the overall accuracy of the algorithm of 59% (95% CI = 0.44-0.73). The sensitivity of the algorithm was low except for the excellent category (0.95). Specificity was moderate to high for good (0.81), limited (0.98), and poor (0.95) categories. PPV was low for all categories and NPV was high for all categories except the good category. Additional results including weighted kappa and inaccuracy of predictions to be presented. DISCUSSION/SIGNIFICANCE OF FINDINGS: PREP2 algorithm, with clinical measures only, is better than chance (chance = 25% for each of the 4 categories) alone at predicting a category of UL capacity at 3 months post stroke. PREP2 is a simple tool that facilitates evaluation of eventual UL outcome from measures routinely captured after a stroke within most healthcare settings in the US.

